# Early Stages of Metal Corrosion in Coastal Archaeological Sites: Effects of Chemical Composition in Silver and Copper Alloys

**DOI:** 10.3390/ma17020442

**Published:** 2024-01-17

**Authors:** Francesca Boccaccini, Cristina Riccucci, Elena Messina, Marianna Pascucci, Ferdinando Bosi, Luca Aldega, Alessandro Ciccola, Paolo Postorino, Gabriele Favero, Gabriel Maria Ingo, Gabriella Di Carlo

**Affiliations:** 1 Institute for the Study of Nanostructured Materials (ISMN), National Research Council (CNR), SP35d, 9, 00010 Montelibretti, Italy; cristina.riccucci@cnr.it (C.R.); elena.messina@cnr.it (E.M.); marianna.pascucci@cnr.it (M.P.); gabrielmaria.ingo@cnr.it (G.M.I.); 2 Department of Earth Sciences, Sapienza University of Rome, Piazzale Aldo Moro, 5, 00185 Rome, Italy; ferdinando.bosi@uniroma1.it (F.B.); luca.aldega@uniroma1.it (L.A.); 3 Department of Environmental Biology, Sapienza University of Rome, Piazzale Aldo Moro, 5, 00185 Rome, Italy; alessandro.ciccola@uniroma1.it (A.C.); gabriele.favero@uniroma1.it (G.F.); 4 Department of Physics, Sapienza University of Rome, Piazzale Aldo Moro, 5, 00185 Rome, Italy; paolo.postorino@uniroma1.it

**Keywords:** silver alloys, copper alloys, alloying elements, corrosion products, burial treatment

## Abstract

In this study, metal disks with different chemical composition (two Ag-based alloys and three Cu-based alloys) were buried in the soil of coastal archaeological sites for a period of 15 years. The aim was to naturally induce the growth of corrosion patinas to obtain a deeper insight into the role of alloying elements in the formation of the patinas and into the degradation mechanisms occurring in the very early stages of burial. To reach the aim, the morphological, compositional and structural features of the patinas grown over 15 years were extensively characterized by optical microscopy, field emission scanning electron microscopy coupled with energy dispersive spectrometry, X-ray diffraction and micro-Raman spectroscopy. Results showed that the Cu amount in Ag-based alloys strongly affected the final appearance, as well as the composition and structure of the patinas. Corrosion mechanisms typical of archaeological finds, such as the selective dissolution of Cu, Pb and Zn and internal oxidation of Sn, occurred in the Cu-based alloys, even if areas enriched in Zn and Pb compounds were also detected and attributed to an early stage of degradation. In addition, some unusual and rare compounds were detected in the patinas developed on the Cu-based disks.

## 1. Introduction

Copper and Ag were extensively used in the past to produce common objects or precious items. Alloying elements were commonly added to the pure metals in order to improve the mechanical properties of the final artifacts or their workability. Silver-based artifacts are usually characterized by the unintentional presence of Pb coming from the extraction processes and by the addition of small amounts of Cu, whereas Cu-based objects were commonly alloyed with Sn, Pb or Zn [1,2]. The nature and the content of the alloying elements, as well as the environmental conditions to which the objects are subjected, strongly affect the corrosion behavior of metal artifacts [3]. Specifically, chlorides severely attack Ag-based and Cu-based alloys, resulting in the growth of corrosion products like silver chloride [4,5], copper chloride [6,7,8] and sometimes lead–chloride compounds [9].

To understand the complex degradation mechanisms that lead to the formation of corrosion layers (i.e., the patina) in burial conditions, archaeometric studies have focused on the characterization of archaeological finds and precious information about the effect of long-term exposure to soil has been obtained [5,6,7,10]. Due to the prolonged interaction with sediment, the metal surfaces spontaneously convert into mineral compounds, usually showing a multilayered structure rich in soil components [11,12]. The high concentration of chlorides and the soil ability to retain water were found to strongly increase the corrosiveness of the burial environment [13,14], while copper dissolution (i.e., decuprification) and the internal oxidation of Sn were recognized as main corrosion patterns occurring in bronze artifacts [15,16,17].

However, the effects of the burial conditions and of the alloy composition on the corrosion behavior cannot be exactly differentiated without a properly designed experiment or without relying on a large set of archaeological samples, and there are still only a few studies that have been conducted using this approach [14,15,18,19]. For instance, a recent work showed that a heterogeneous distribution of chlorides can occur within the same archaeological site, leading to a different corrosion extent of the buried objects [20]. Moreover, information about the early stages of degradation processes in burial conditions cannot be acquired when archaeological finds are considered, since these are the results of hundred years of metal/soil interactions.

A few attempts to study the corrosion behavior of metal substrates for a short permanence time in soil (i.e., about one year) were made [21,22]. These studies focused on examining the composition and the morphology of patinas grown on bronze disks, which were chemically pretreated with CuCl_2_ and NaCl solutions and then buried for about one year in the soil of Tharros (Western Sardinia coast, Italy). Interesting results were obtained such as the formation of copper corrosion products, typical of archaeological objects (i.e., copper hydroxychlorides), was successfully induced [21] and the Cu migration from the bulk to the external environment was observed [22]. However, the effect of the alloying elements on the composition and structure of corrosion patinas was not deeply investigated. The behavior of different alloys in the first stages of burial is still an open question.

It is worth mentioning that studying the effect of a short-term burial (i.e., a few years) on Ag-based and Cu-based alloys and comparing the results with those observed on long-term buried artifacts (i.e., hundred years) is also important from a conservation point of view. Indeed, artificial patinas produced in short-term experiments and showing structural and chemical features similar to those of ancient and artistic objects can be used as reference substrates to perform validation tests of novel cleaning or protective materials in place of the precious and unique artifacts [23,24,25,26,27].

In this work, a large number of metal disks with different chemical composition were intentionally buried for 15 years in the soil of archaeological sites to identify the effect of the alloying elements in the formation of corrosion patinas and to investigate the first stages of degradation. To this purpose, two Ag-based and three Cu-based alloys with microchemical and microstructural features similar to those of ancient metals were selected and buried into the soil of the archaeological sites of Tharros and Sant’Antioco (Western Sardinia coast, Italy), both in situ and ex situ. These were ancient Phoenician settlements, later conquered by Carthaginians and Romans, located in the western Sardinia coast. The proximity to the sea makes the soil of these areas highly aggressive due to large amounts of chlorides, particularly dangerous for metal artifacts composed of Ag, Cu and Fe [28,29]. For this reason, the sites were carefully chosen to conduct the in situ burial treatment and samples of soil were collected and used for the ex situ laboratory experiments. Once excavated, the morphological, compositional and structural features of the patinas grown on the metal disks were deeply investigated by optical microscopy (OM), field emission scanning electron microscopy coupled with energy dispersive spectrometry (FE-SEM-EDS), X-ray diffraction (XRD) and micro-Raman spectroscopy. The results were critically compared, also considering the properties and mechanisms of formation of archaeological patinas formed over a long-term burial. The amount of Cu in the Ag-based alloys was found to affect the final appearance and composition of the patinas, whereas the corrosion mechanisms typical of archaeological Cu-based artifacts (i.e., decuprification, dezincification, loss of Pb and internal oxidation of Sn) were also observed in this study, even if at an early stage. Some rare and uncommon compounds were also found on the Cu-based disks.

## 2. Materials and Methods

### 2.1. Ag-Based and Cu-Based Alloys

Two Ag-based alloys and three Cu-based alloys were selected for the experiments as representative of the ancient metal artifacts, both in terms of chemical composition and metallurgical features. The alloys were produced following existing procedures [21,30]. A dendritic microstructure, typical of casted metals, was obtained by the rapid cooling of the molten metal, whereas different chemical compositions were chosen to include and reproduce all the major families of archaeological alloys (i.e., silver, bronze, leaded bronze, brass). Disks with a diameter of 18 mm and a thickness of about 2 mm were obtained for the Ag-based alloys, whereas disks with a diameter of 24 mm and a thickness of 3 mm were obtained for the Cu-based alloys. In Table 1, a summary of the chemical composition of each alloy, with information about their use in the paste, is reported.

### 2.2. Experimental Conditions

The reference disks (50 in total) were intentionally buried in soil for about 15 years to promote the growth of corrosion patinas. Specifically, the two Ag-based alloys and the bronze one were directly buried in situ in the archaeological site of Tharros (western Sardinia coast, Italy) at a depth of about 30 cm from the ground surface, the leaded bronze was buried in the Tharros’ soil in laboratory (ex situ), while the brass was buried ex situ in the soil of both Tharros and Sant’Antioco (western Sardinia coast, Italy). Once unearthed, the disks were gently washed with distilled water to remove soil particles. The 15-year-long burial treatment was selected as the duration was considered enough to promote the natural growth of corrosion patinas and was compatible with the research timeframe.

In the case of ex situ experiments, the sediment was initially mixed with NaCl (4% *v*/*v*), and distilled water was periodically poured into the containers, filled with the soils, where the disks were inserted, in order to simulate the action of rain. Holes were made on the bottom of the containers to let the water flow away and avoid stagnation.

In a previous work, we reported that in situ and ex situ burial treatments carried out for the same time (i.e., about 15 years) led to similar results in terms of structure and composition of the corrosion patinas, except for surface roughness [31]. Therefore, any difference in the nature of the corrosion products and in the corrosion behavior of the five alloys was mainly related to their specific chemical composition and microstructural features. In addition, the effect of the soil typology (i.e., Tharros and Sant’Antioco) was evaluated by comparing the brass samples buried ex situ in both the sediments. 

For each alloy composition, a representative sample was selected and described in this paper.

### 2.3. Structural, Microchemical and Mineralogical Analysis

In order to analyze the morphological features of the corrosion patinas, samples were initially observed using a Leica MZFLIII optical microscope and a Leica application suite (LAS) multifocus stereo microscope, both equipped with a digital camera (Leica DFC 320).

The micro-structure and the micro-chemical information were obtained using a high-brilliance and high-spatial-resolution LEO Gemini 1530 (Zeiss, Oberkochen, Germany) field emission scanning electron microscope, coupled by an energy dispersive X-ray spectrometer INCA 450 and four-sector back-scattered electron detectors. Images were all recorded in the back-scattered mode at different acceleration voltages from 1 to 20 kV.

The mineralogical assemblage of soils, of patinas grown on Ag-based disks and of powders scratched from the patinas of copper-based alloys were investigated using powder X-ray diffraction analysis conducted with a Bruker D8 Advance X-ray system equipped with Lynxeye XE-T silicon-strip detector at the Department of Earth Sciences, Sapienza University of Rome (Italy). The X-ray system was operated at 40 kV and 30 mA using CuKα radiation (λ = 1.5406 A°). Samples were run from 2° to 70° 2θ with a step size of 0.02° 2θ while spinning the sample. Data were collected with variable slit mode to keep the irradiated area on the sample surface constant. Diffractograms were analyzed by an electronic database (DIFFRAC.EVA, version 5.2).

The mineralogical composition was also studied by micro-Raman spectroscopy using a Horiba Jobin Yvon HR-Evolution micro-Raman spectrometer equipped with a 633 nm excitation laser and dry objectives. Acquisitions in two ranges (125–1295 and 1045–2085 cm^−1^) were obtained. Specific acquisition settings were chosen for each sample depending on degradation sensitivities and fluorescence, and parameters were optimized for every spectrum; magnifications were 20× or 100×; maximum laser power at the source: 15 mW; accumulation time between 5 s and 60 s; and number of scans was between 10 and 30. Samples were placed directly on a microscope slide for analysis.

To study the corrosion mechanisms, cross sections were realized by using a diamond saw and by embedding samples’ fragments in epoxy resin for 24 h. Sections were polished with silicon carbide papers until 1200 grit and diamond pastes up to 0.1 µm. Optical analysis of the cross sections was performed by the Leica MEF IV optical microscope equipped with a 420 Leica digital camera. Microchemical and microstructural analyses were performed by the FE-SEM-EDS equipment already described and by a field emission scanning electron microscope MAGNA (Tescan, Brno, Czech Republic) equipped with an energy dispersive X-ray spectrometer AZtec ULTIM MAX 65 (Oxford, UK). Before the analysis, the surfaces were coated with a thin layer of carbon to avoid the charging effects induced by the electron beam. Carbon layers were deposited by using a Bal-Tech SCD 500 apparatus equipped with a turbo pumping system for ultraclean preparations at a pressure of 5 × 10^−3^ mbar to produce a conductive film with a uniform thickness of about 1.0 nm.

## 3. Results and Discussion

Table 2 shows the average chemical composition of the soils obtained using EDS analysis, whereas the powder XRD diffraction patterns are shown in the Supplementary Material section. The pH of the two soils was previously measured and reported elsewhere [29]. The pH value is 8.7 and 8.5 for the soil of Tharros and Sant’Antioco, respectively.

The site of Tharros is situated on Pleistocene deposits mainly composed of aeolian sandstones rich in vertebrate bones (sheet 528—Oristano, scale 1:50,000, ISPRA, in press [32]). The representative XRD pattern of the soil developed on the Pleistocene deposits displays quartz, carbonate minerals such as calcite and aragonite, K-feldspar, albite, sheet silicate minerals (muscovite, kaolinite, chlorite) and halite (Figure S1). The presence of halite was attributed to the crystallization of the mineral from marine aerosols containing Na and Cl ions. The site of Sant’Antioco is located on Miocene pyroclastic deposits with rhyolitic composition (sheet 564—Carbonia, scale 1:50,000, ISPRA, in press [33]). As evidenced by the XRD pattern (Figure S2), the soil formed at the expense of pyroclastic deposits contains quartz, calcite, aragonite, K-feldspar, albite, muscovite, kaolinite, chlorite and smectite. The occurrence of smectite is typical of the chemical weathering of the volcanic glass of pyroclastic deposits [34]. 

**Figure 1:**
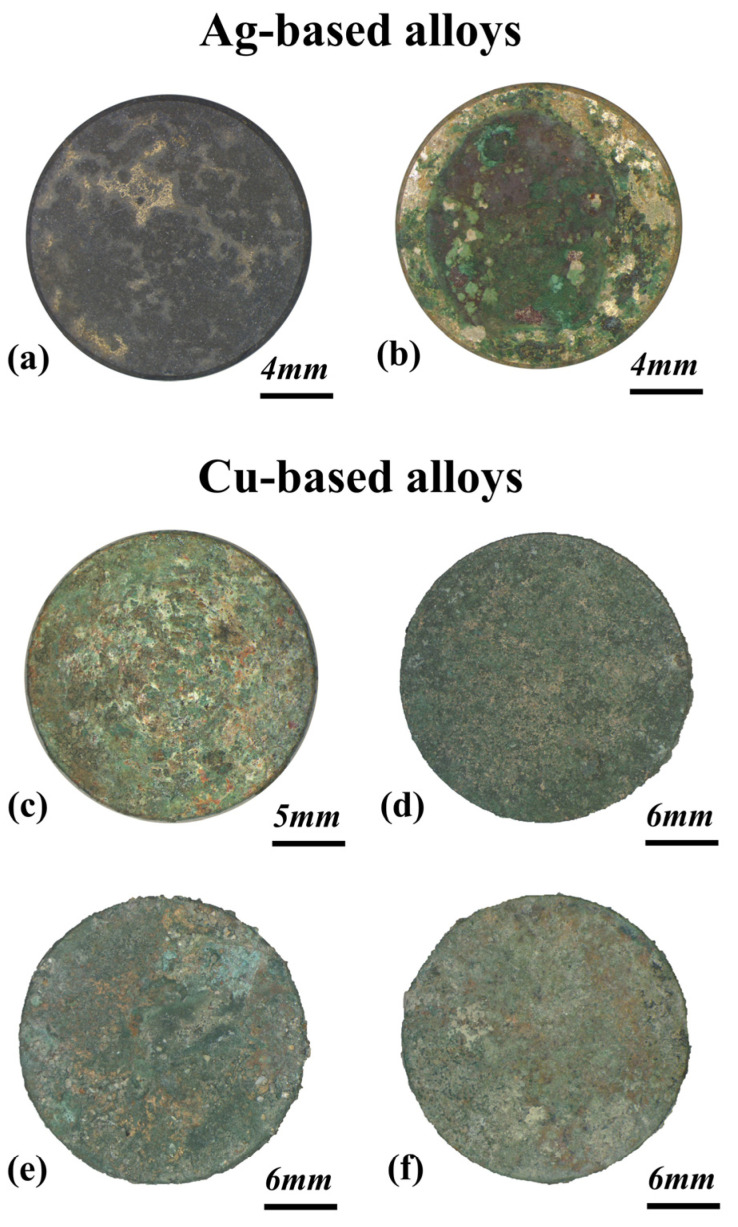
Surface appearance of the six disks after 15 years of burial. Optical images show the color and the general appearance of the corrosion patinas grown on the Ag-Cu6.5 (**a**), the Ag-Cu7.5 (**b**), the bronze (**c**), the leaded bronze (**d**) and the brass disks, buried respectively in the soil of Sant’Antioco (**e**) and Tharros (**f**).

Figure 1 shows the appearance after excavation of the most representative corroded disks, both composed of Ag-based and Cu-based alloys. The 15-year-long burial led to the development of well-formed corrosion patinas on all the disks. The original metal surfaces converted into colorful mineral compounds, as a result of the prolonged interaction with soil.

### 3.1. Silver-Based Alloys

The corrosion behavior of Ag-Cu alloys is highly influenced by the metallurgical features. Casted silver artifacts containing Cu show a dendritic microstructure with phase separation resulting from the limited solid-state solubility of the two elements [1]. During the rapid cooling, nearly pure copper islands separate from the silver-enriched matrix and solidify in the space among the Ag dendrites. The heterogeneous structure gives rise to the galvanic coupling between the nobler silver areas and the anodic copper islands, activating electrochemical cells and promoting corrosion. The Cu content in the alloy is then a key factor in determining the extent and the rate of degradation, also influencing the type of corrosion compounds [35].

As evidenced by optical images of Figure 1, a flat and compact patina grew on the Ag-Cu6.5 disk. The surface appears to be dark gray overall, with few brighter areas corresponding to the underlying metal layer. In contrast, the patina developed on the Ag-Cu7.5 sample exhibits a more complex and rougher surface. Green corrosion compounds formed over an underlying layer that is made of brown alteration products (details in Figure S3). Sediment grains are also present in this patina.

**Figure 2:**
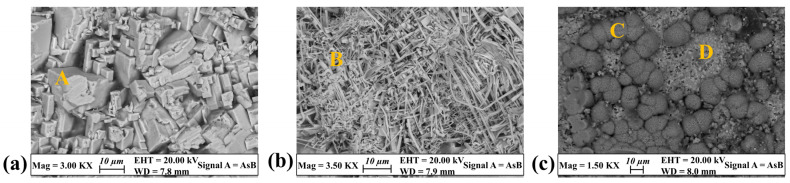
Representative FE-SEM backscattered images showing the habit of the corrosion compounds grown on the silver-based substrates. Crystals with a squared (**a**) up to fibrous (**b**) habit developed on the surface of the Ag-Cu6.5 disk, while needle-like compounds forming rounded aggregates grew over a layer of micrometric crystals on the Ag-Cu7.5 sample (**c**). Yellow capital letters indicate the areas where EDS analyses have been acquired (see Table 3).

In order to deeply investigate the corrosion behavior of the two silver-based disks, micro-structural and micro-chemical surface analyses were carried out with FE-SEM-EDS. The results revealed that differences in terms of color and structure are related to the nature and chemical composition of the corrosion compounds, and that Ag-based alloys are extremely sensitive to the presence of chlorides in the surrounding environment. More in detail, FE-SEM-EDS analysis acquired in correspondence of the dark gray areas of the Ag-Cu6.5 patina pointed out that the surface is composed of well-formed micrometric crystalline compounds containing Ag and Cl (Figure 2a,b and Table 3 (A,B)). These alteration products are spread all over the surface and present different crystal habits. They crystallized both as squared and as columnar (up to fibrous) compounds, showing cubic symmetry as evidenced by the direction of cleavage. 

On the other hand, the corrosion patina grown on the Ag-Cu7.5 disk is mainly composed of copper alteration products (Figure 2c and Table 3 (C,D)). FE-SEM-EDS analysis, acquired on representative green areas, revealed that two different Cu-containing compounds developed on the surface. Crystals with a needle-like habit and clustered in botryoidal aggregates are composed of Cu, O and C, probably consisting of copper carbonates (Table 3 (C)). Granular micrometric compounds are instead composed of Cu, Cl and O, suggesting the presence of copper hydroxychlorides (Table 3 (D)). Moreover, small amounts of elements coming from the soil, like Si and Ca, were detected among the copper corrosion products.

**Figure 3:**
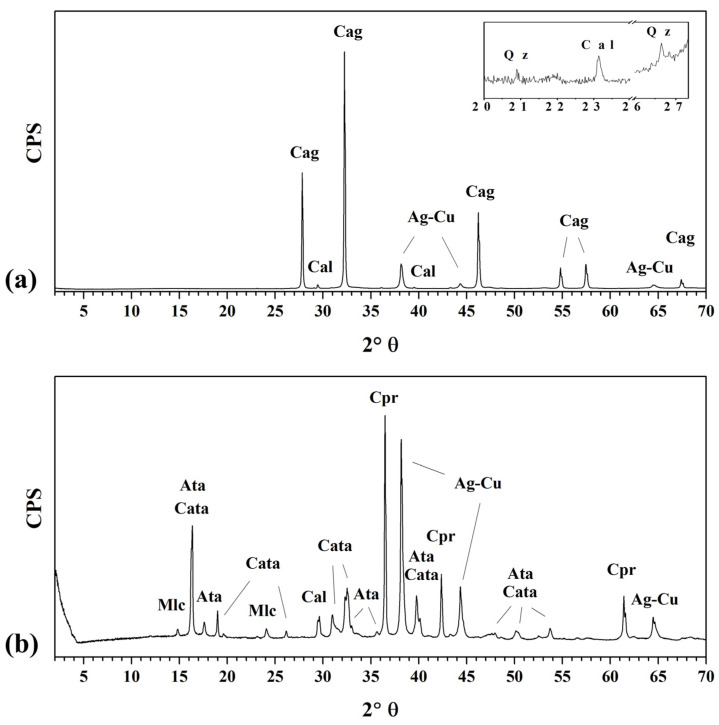
XRD patterns showing the mineralogical assemblage of the corrosion products grown on the two silver-based alloys. The X-ray pattern acquired on the surface of the Ag-Cu6.5 disk (**a**) shows chlorargyrite (Cag) and minerals such as calcite (Cal) and quartz (Qz), while the one acquired on the Ag-Cu7.5 sample (**b**) shows cuprite (Cpr), malachite (Mlc), atacamite (Ata), clinoatacamite (Cata) and calcite (Cal). The inset in graph (**a**) shows a magnification of the X-ray pattern in the region from 20° to 27° 2θ. Reflections at 38.2°, 44.3° and 64.5° 2θ were attributed to the underlying metal alloy (Ag-Cu). Attributions were made using the following JCPDS codes: 06-0480 (Cag), 83-1762 (Cal), 05-0490 (Qz), 77-6540 (Ag-Cu), 78-2076 (Cpr), 56-0001 (Mlc), 25-0269 (Ata) and 50-1559 (Cata).

To better identify the mineralogical assemblage of the corrosion compounds, XRD patterns were acquired on the surface of each silver-based disk. The patinas are composed of degradation products that typically form on archaeological silver objects over a hundred years of burial [5,35,36]. The XRD patterns, reported in Figure 3, confirmed that an Ag-containing mineral (i.e., chlorargyrite (AgCl)) developed as major component in the patina of the Ag-Cu6.5 sample, whereas only Cu-containing corrosion compounds, specifically cuprite (Cu_2_O), malachite (Cu_2_(CO_3_)(OH)_2_), atacamite and clinoatacamite polymorphs (Cu_2_Cl(OH)_3_), formed on the other alloy. As a result of the prolonged interaction with the soil, sediment grains, composed of calcite and quartz, were also detected in the patinas.

The analysis of the cross-section samples provides information on the composition and stratigraphy of corrosion layers and allowed us to better understand the degradation mechanisms, which can be explained considering the decuprification phenomenon and the evolution of the cuprite layer during the alloy oxidation. At the very early stages of corrosion, the reactive and less noble copper islands preferentially oxidize and convert into cuprite. This oxide layer can later react with the anions dissolved in the surrounding soil (such as Cl^−^, HCO_3_^−^, SO_4_^2−^, PO_4_^3−^) and progressively turn into green Cu(II) corrosion compounds. Some of the Cu can also be leached from the surface toward the ground. In support of this explanation, FE-SEM-EDS analysis of the cross section obtained from the Ag-Cu7.5 disk revealed a double-layered stratigraphy, with a thickness of about 15–20 μm and consisting of an inner cuprite layer under Cu(II) minerals (Figure S4a). However, with a lower Cu amount in the silver alloy, it seems that all the shallow copper islands dissolve and leach into the ground, without forming a stable cuprite layer on the disk surface. The analysis of the cross section of the Ag-Cu7.5 sample showed that the corrosion patina is exclusively made of chlorargyrite, forming a thin and fractured layer (about 10 μm) over the metal core (Figure S4b). Neither cuprite nor Cu(II) compounds were detected in this sample.

These findings suggest that differences in corrosion behavior, i.e., in the formation of Ag- or Cu-bearing corrosion products, are related to the chemistry of the original alloy, especially to the Cu amount. Small variations in the Cu content (about 1%) led to major differences in terms of color, structure and composition of the corrosion patinas. According to these results, previous archaeometric studies detected chlorargyrite as a single corrosion product on archaeological Ag-based objects with a Cu amount lower than 6.5% [35,37]. The Cu content in the silver alloy thus seems to be a driving factor in determining the typology of compounds that developed during corrosion.

### 3.2. Copper-Based Alloys

**Figure 4:**
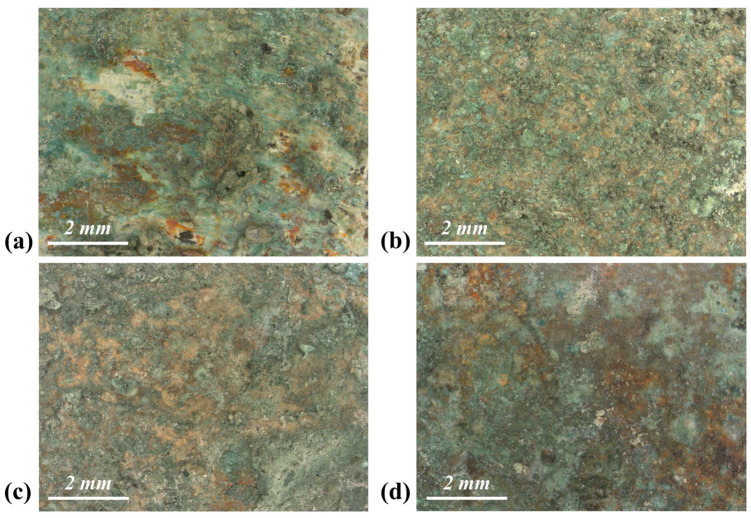
Optical images showing the morphological features of the corrosion patinas grown on the copper-based alloys. Green compounds mixed with sediment grains developed on the surface of the bronze (**a**), the leaded bronze (**b**) and the brass disks buried in the Sant’Antioco (**c**) and Tharros (**d**) soil.

As shown in Figure 1, a green corrosion patina developed on the surface of the Cu-based alloys. At higher magnification (Figure 4a), the patina of the bronze alloy appears quite heterogeneous. In some areas, the green compounds fractured, disclosing underlying flat orange or whitish layers. Black arborescent phases are also widely spread on the surface (details in Figure S5). The patina developed on the leaded bronze is more homogeneous and has a dusty appearance. When observed at higher magnification (Figure 4b), the green layer appears discontinuous and formed by rounded aggregates grown over an underlying orange layer (details in Figure S6). The surface of the brass samples also presents a pronounced orange layer, above which green compounds developed (Figure 4c,d). Sediment encrustations were detected on all the samples.

The mineralogical assemblage of the corrosion patinas was investigated by XRD analysis acquired on the powders scratched from each surface. X-ray patterns show that the four patinas are mainly composed of copper corrosion products (Figures S7–S10). Copper oxide (cuprite) and copper hydroxychlorides (atacamite and clinoatacamite) were detected in all the samples, whereas malachite was found only on the leaded bronze disk and on the brass sample buried in the Sant’Antioco soil. In addition to copper compounds, a tin corrosion product, namely schoenfliesite (MgSn(OH)_6_), was found in the bronze and leaded bronze disks, as well as in the brass sample buried in the Tharros soil. Clastic minerals from the sediment such as quartz and calcite were also ubiquitously detected.

**Figure 5:**
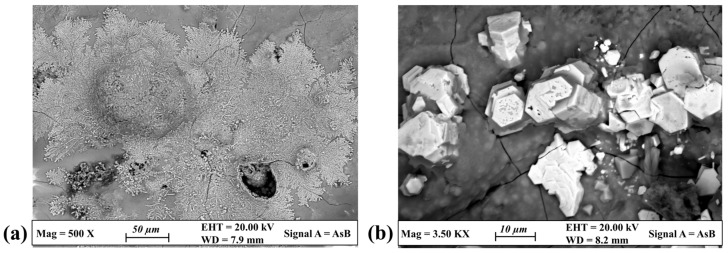
Morphological features of minor compounds found on the patinas. FE-SEM backscattered images showing the occurrence of arborescent phases on the surface of the bronze patina (**a**) and of hexagonally shaped crystals on the leaded bronze disk (**b**).

Complementary information on compounds was achieved by FE-SEM-EDS analysis. FE-SEM images of Figure 5 show the morphological features of two compounds that were locally found on the surface of the patinas. Granular nanocrystals aggregated to form arborescent structures occur on the surface of the bronze disk (Figure 5a). As revealed by EDS analysis, summarized in Table 4, they are composed of Cu and S, suggesting the presence of some copper sulfides. The thin sulfide layer lays on other copper corrosion compounds, specifically copper hydroxychlorides, as evidenced by the presence of relatively small amounts of Cl and O. In the literature, there are many occurrences of copper sulfide naturally grown on copper-based artifacts which have been buried for a long time in soil [38,39] or aqueous sediments [40,41]. It is known that they form under anoxic conditions in the presence of decomposing organic matter and sulfate-reducing bacteria (SRBs). These microorganisms can convert sulfate ions into corroding H_2_S, thus promoting the growth of various copper sulfides with stoichiometry ranging from CuS (covellite) to Cu_2_S (chalcocite) [2].

On the other hand, the hexagonally shaped crystals developed on the leaded bronze disk (Figure 5b) are much rarer. They are mainly composed of Pb along with Na and O. Small Cu amounts were also detected and are probably related to copper corrosion products deposited on the crystals, which appear altered in the FE-SEM image. Some occurrences of sodium lead carbonate developed on artistic objects were recently reported [42]. The compound was found in the form of a white pigment and as a corrosion product, exhibiting a hexagonal shape, on a leaded foil that had been exposed to an Na_2_CO_3_ solution. Additionally, it was observed on glass beads that were coated with molten lead [43]. In the latter case, a degradation process was induced by the glass, which released sodium into the surrounding environment. Concurrently, abellaite, a new mineral of hexagonal symmetry and ideal formula NaPb_2_(CO_3_)_2_(OH), was discovered [44]. It commonly forms crystals with a tabular to lamellar habit, grouped into disordered aggregates. Considering all this information and by combining the morphological data obtained by FE-SEM investigations with the chemical composition revealed by EDS analysis, the hexagonally shaped crystals developed on the leaded bronze disk suggest the presence of abellaite mineral in the patina. The sodium chloride from Tharros soil (naturally present or added) could have reacted with the Pb ions coming from the corrosion of the alloy and with carbonate species or environmental CO_2_ dissolved into the water, thus resulting in the formation of such a rare corrosion product.

**Figure 6:**
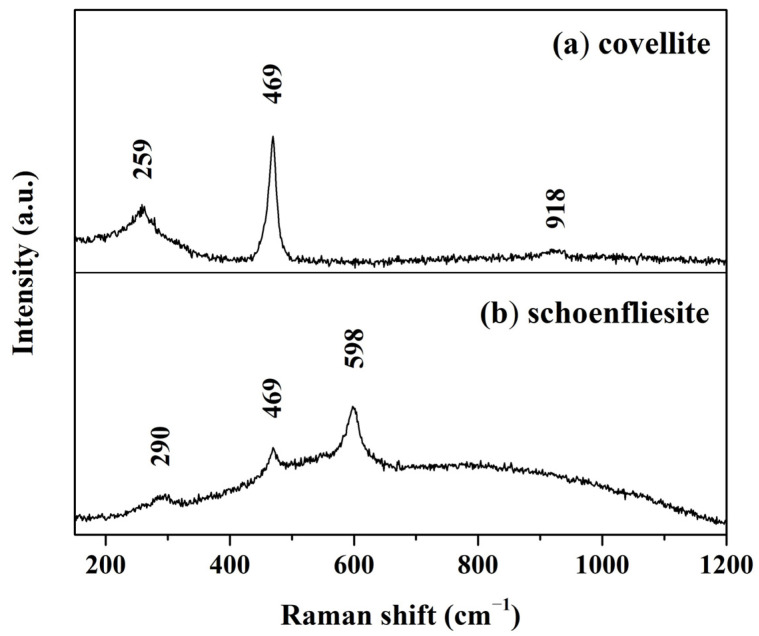
Micro-Raman spectra acquired on the black arborescent (**a**) and on the flat white (**b**) compounds developed on the bronze disk. Spectra were attributed to covellite and schoenfliesite minerals, respectively.

Since the sulfide arborescent phases were also easily detected by optical microscopy, as they form black and shallow layers on the patina surface (Figure S5), micro-Raman analysis was used to enhance the understanding of their mineralogy. The spectrum, collected on the surface of an arborescent compound, is shown in Figure 6a. In accordance with the literature, the compound was identified as covellite (CuS) [45,46]. The main band at 469 cm^−1^ and the minor at 259 cm^−1^ are related to the vibrational mode of Cu–S bond [47], while that at 918 cm^−1^ was attributed to a second-order Raman scattering [48]. However, as many stable copper sulfides are known [2], the occurrence of other Cu_x_S_y_ phases cannot be ruled out.

By combining the information obtained from OM and FE-SEM-EDS, it was also possible to record the Raman spectrum of schoenfliesite (Figure 6b). It was acquired on a flat and white area of the bronze patina, where FE-SEM-EDS analysis detected a significant amount of Sn and Mg (Figure S11). It is worth mentioning that the white area where the spectrum was collected is the same where FE-SEM-EDS analysis detected Sn and Mg. The main peak occurs at 598 cm^−1^, whereas other minor bands were recorded at 469 cm^−1^ and 290 cm^−1^, matching the reference spectrum of synthetic MgSn(OH)_6_ powder [49,50]. To the best of the authors’ knowledge, this is the first time that schoenfliesite has been identified as a bronze corrosion product by means of Raman spectroscopy, since previous occurrences were detected exclusively by X-ray diffraction analysis [31,51].

In order to obtain further insights into the role played by alloying elements in the patina formation and to investigate the early stages of degradation mechanisms, cross-sectioned samples were realized and analyzed. As already reported in our previous work [31], the stratigraphy of the bronze patina developed during 15 years of burial into the Tharros soil consists of a 20–30 μm thick layered structure formed by copper hydroxychlorides mixed with sediment grains and developed over Sn and Mg compounds, which in turn formed over an inner layer of copper oxide (Figure S12). This structure has been explained by the copper preferential oxidation and dissolution into the ground, followed by the precipitation of Cu(II) corrosion products, and by the conversion of Sn into tin oxides (or hydroxides) growing from the surface toward the core. Though stable, tin compounds are also very brittle, forming micro-cracks through which oxygen and aggressive ions can penetrate and promote further corrosion of the metal alloy.

**Figure 7:**
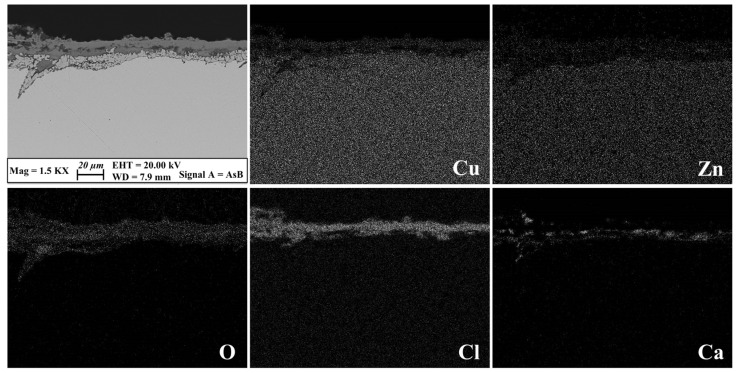
Structural and chemical features of the cross-sectioned patina grown on the brass disk buried ex situ in the Sant’Antioco soil. FE-SEM backscattered image and relative X-ray elemental maps show a two-layered corrosion patina. An inner layer composed of Cu and O and depleted in Zn developed above the metal core, whereas the outermost corrosion products are mainly Cu and Cl compounds. The presence of Ca among the corrosion layers indicates calcite grains within the patina.

**Figure 8:**
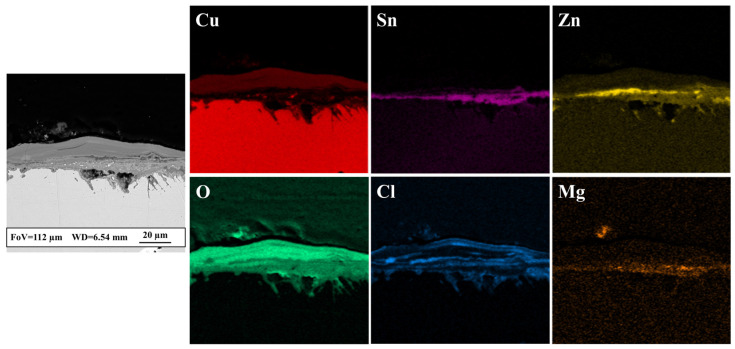
Structural and chemical features of the cross-sectioned patina grown on the brass disk buried ex situ in the soil of Tharros. FE-SEM backscattered image and relative X-ray elemental maps show a layered corrosion patina, revealing the presence of tin compounds, mostly associated with Mg. The outermost patina is mainly composed of Cu and Cl corrosion products, whereas Zn-containing compounds form a layer just above the original surface bound.

The loss of zinc from the metal surface (i.e., dezincification process) is known to be the mechanism of corrosion of brass alloys [2]. Analysis of cross-sectioned patinas showed that the Zn depletion occurs at the interface between the original bound of the object and the external corrosion layers [52,53,54]. Due to the tendency of zinc to diffuse into the soil, Zn-containing corrosion products, like zincite (ZnO) and zinc chloride hydroxide (Zn(OH)Cl), were seldom detected on brass patinas [55]. In Figure 7, a representative FE-SEM image with relative X-ray elemental maps acquired on the cross section obtained from the brass disk, buried in the soil of Sant’Antioco, is reported. The patina is about 20 μm thick and it is mainly composed of two layers: an inner layer formed by Cu and O (i.e., cuprite) and a superimposed one formed by Cl species (i.e., copper hydroxychlorides) and sediment grains (i.e., calcite). The inner corrosion layer is clearly depleted in Zn due to the dezincification process, though the element is unevenly diffused among the outermost patina compounds. However, in some areas, especially those with a swelled appearance, the Zn content reaches up to 20–30% by weight (Figure S13). The presence of high amounts of the element, apparently in contrast with the dezincification process, can be ascribed to the relatively short period of burial to which the brass disk was subjected. During this stage of patina formation, it is likely that insufficient time had passed for the complete depletion of Zn, resulting in its precipitation as a corrosion compound after oxidation. In addition, the presence of a superimposed layer of copper products acting as a physical barrier could have hindered Zn diffusion toward the outside. It is worth noting that the occurrence of Zn-enriched areas in the patina was also detected in the brass sample buried in the Tharros soil (Figure 8).

As shown by XRD analysis, the main difference between the brass disks buried in the Sant’Antioco and in the Tharros soils is the presence of tin compounds, such as schoenfliesite. The FE-SEM-EDS analysis of the cross section obtained from the sample buried in the Tharros soil confirmed that a Sn-enriched layer, mostly combined with Mg, developed in correspondence of the original surface edge (Figure 8). The occurrence of schoenfliesite could be due to higher amounts of Mg in the soil of Tharros (Table 2), which would favor the formation of the mineral. However, the element content between the two sediments only varies by a few tenths of percentage, and this difference is too low for effectively supporting the latter hypothesis. Further data about corrosion products of buried brasses would thus be statistically meaningful to deeply investigate the relationship between schoenfliesite and Mg content in soil. It is worth mentioning that the occurrence of Sn-enriched layers in brass objects is important from a conservation point of view, as Sn is known to enhance the corrosion resistance of Cu-Zn alloys [52].

**Figure 9:**
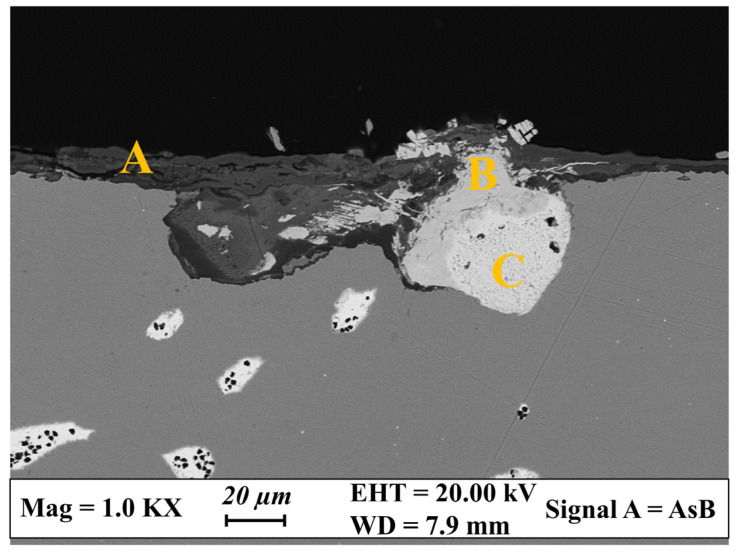
Structural features of the cross-sectioned patina grown on the leaded bronze disk. FE-SEM backscattered image shows the stratigraphy of the corrosion compounds. Yellow capital letters indicate the areas where EDS analyses have been acquired (see Table 5).

During the production phases, Pb has usually been added to Cu and the other alloying elements with the aim of increasing the fluidity of the molten metal and of maintaining low production costs [2]. However, the solubility of Pb in Cu is extremely low and, once solidified, Pb forms globules dispersed in the copper matrix [1,18,56,57]. These areas show an anodic behavior during corrosion and preferentially oxidize. In Figure 9, a representative FE-SEM image of the cross section obtained from the leaded bronze disk is reported, while the relative EDS analyses are provided in Table 5. The patina appears heterogeneous in terms of thickness and composition, as well as more heavily corroded with respect to those developed on the other copper alloys. A thin corroded layer, with a thickness of about 10–20 μm, was found in correspondence to the surface original bound (point A). It contains Cu, Sn, Cl, Si, C and O, suggesting the presence of copper hydroxychlorides, copper carbonates and tin hydroxides mixed with sediment clasts, as already pointed out by XRD analysis. An area with a patina’s thickness of more than 50 μm and mainly composed of Pb-containing compounds was also detected in the same FE-SEM image (points B and C). These compounds replaced the space originally filled by the lead globules, supporting the hypothesis that corrosion occurs toward the bulk along the lead islands [58]. The EDS results suggest the formation of lead chlorides and mixed sodium–lead carbonates. The occurrence of lead chlorides at the interface with the metal core confirms that chloride ions are very aggressive toward Cu-Pb alloys and that they can easily penetrate the inner layers favored by the anodic dissolution of the alloying elements [9,59,60].

The presence of a big corroded area composed of Pb-containing compounds at the interface with the metal core was attributed to an early stage of lead dissolution, that is a typical corrosion mechanism observed on artifacts buried for long-term periods in soil. Lead usually oxidizes and migrates toward the soil, leaving the areas underneath the original surface bound depleted as corrosion proceeds [58], even if Pb compounds were sometimes found in the external corrosion layers [60,61]. However, in our leaded bronze disk, the time was not long enough to promote the total dissolution and migration toward the outside of the lead corrosion products, which still form a thick layer at the interface with the metal core. With time, they would totally dissolve and diffuse into the soil, as suggested by some Pb-based compounds that were found in the shallow layer of the patina (Figure 5b and Figure 9).

Overall, the production of specific alloys with compositional and metallurgical features similar to the ancient ones and the 15-year-long burial have been successful in reproducing corrosion patinas typical of archaeological finds. The extensive characterization of the morphological, compositional and structural features of the buried disks pointed out that these samples are similar to the most common Ag-based and Cu-based corroded artifacts. For this reason, they can be used as representative materials to conduct reliable validation studies. The production of patinas with different structure and composition indeed provided a set of mock-ups that can act as disposable substrates for the validation of novel conservation treatments in place of the nobler and unique works of art.

## 4. Conclusions

In this study, several metal disks with different compositions were buried for 15 years in the soil of coastal archaeological sites to evaluate the effects of the alloying elements on the corrosion patinas and to investigate the early stages of degradation.

The results pointed out that the Cu content in Ag-based disks highly affected the appearance as well as the chemical and mineralogical composition of the patinas developed on the surface. A gray patina composed of silver compounds (i.e., chlorargyrite) formed on the alloy with minor Cu content, whereas only Cu-based compounds (i.e., cuprite, malachite, atacamite and clinoatacamite), providing a green appearance to the surface, developed on the disk with the higher Cu amount.

The patinas grown on the Cu-based disks show a general green appearance, with local morphological features specific for each sample. The mineralogical assemblage of the four patinas was found to be similar. Copper corrosion products (i.e., cuprite, atacamite and clinoatacamite) and minerals coming from the soil (such as calcite and quartz) were detected in all the samples. Some minor and rare compounds were also detected, such as covellite, schoenfliesite and a sodium and lead carbonate forming hexagonally shaped crystals tentatively assigned to abellaite mineral. The investigation of cross-section samples suggested that the corrosion mechanisms typical of copper-based alloys (i.e., decuprification, dezincification, internal oxidation of Sn and preferential depletion of Pb) also occur in the early stages of degradation in soil. Some exceptions to these trends, such as the presence of corroded areas highly enriched in Zn- or Pb-containing compounds, were ascribed to the relatively short time of growth of the patinas, whose formation processes were still ongoing at the time of excavation.

The soil type (i.e., Tharros and Sant’Antioco soil) did not significantly affect the appearance of the brass patinas, although a difference in the composition (i.e., in the formation of schoenfliesite) was observed.

Overall, the feasibility of producing archaeological-like corroded mock-ups was proven. The 15-year-long burial successfully induced the growth of corrosion patinas typical of Ag-based and Cu-based artifacts. The nature and the amount of alloying elements played a key role in the patinas’ composition and structure.

The experimental procedure and the mock-ups’ characterization conducted in this work represent a valuable starting point for further investigation about the artificial reproduction of archaeological finds, also with the perspective of reducing the burial duration.

## Figures and Tables

**Table 1:** Nominal chemical composition of the reference alloys, expressed in wt%.

	Ag	Cu	Pb	Sn	Zn
Ag-Cu6.5	92.0	6.5	1.5	-	-
Ag-Cu7.5 (monetary use)	92.5	7.5	-	-	-
Bronze (common use objects)	-	92.3	0.2	7.5	-
Leaded bronze (statuary)	-	88	8	4	-
Brass (orichalcum)	-	82.5	0.5	3	14

**Table 2:** Overall chemical composition of the Tharros and Sant’Antioco soil analyzed by EDS. All results are expressed in wt%; n.d. = not detected.

	C	O	Na	Mg	Al	Si	P	Cl	K	Ca	Ti	Fe
Tharros	14.6	46.8	2.3	1.0	4.4	14.7	0.5	2.0	1.7	8.4	0.3	3.3
Sant’Antioco	14.5	48.0	1.3	0.5	6.0	17.6	n.d.	n.d.	3.6	6.2	n.d.	2.3

**Table 3:** Chemical composition of the patinas grown on the Ag-based alloys revealed by EDS analysis, acquired in correspondence of the areas indicated by yellow capital letters in Figure 2. The squared and fibrous crystals (point A and B, respectively), that developed on the Ag-Cu6.5 disk, are composed of silver chlorides, while the compounds formed on the Ag-Cu7.5 disk are copper corrosion products, likely copper carbonates (point C) and copper hydroxychlorides (point D). Elements like Si and Ca from sediment grains were also detected in the Ag-Cu7.5 patina. All results are expressed in wt%; n.d. = not detected.

	C	O	Si	Cl	Ca	Cu	Ag
Squared crystals (A)	n.d.	n.d.	n.d.	16.9	n.d.	n.d.	83.1
Fibrous crystals (B)	n.d.	n.d.	n.d.	17.5	n.d.	n.d.	82.5
Needle-like crystals (C)	12.0	36.9	0.7	n.d.	n.d.	50.4	n.d.
Granular crystals (D)	n.d.	27.3	0.9	16.2	0.6	55.0	n.d.

**Table 4:** Chemical composition, revealed by EDS analysis, of the arborescent phases and hexagonally shaped crystals developed on the bronze and leaded bronze disk, respectively. All results are expressed in wt%; n.d. = not detected.

	C	O	Na	S	Cl	Cu	Pb
Arborescent phases	8.2	4.0	n.d.	13.2	4.4	70.2	n.d.
Hexagonal crystals	8.6	16.7	3.1	n.d.	n.d.	6.0	65.6

**Table 5:** Chemical composition of the leaded bronze patina revealed by EDS analysis, acquired in correspondence of the areas indicated by the yellow capital letters in Figure 9. The outermost corrosion layers are composed of Cu and Sn compounds, formed in correspondence of the original surface bound and mixed with sediment clasts (point A). Pb-containing compounds developed in large amounts at the interface with the metal core (points B and C), replacing the space originally occupied by Pb globules. All results are expressed in wt%; n.d. = not detected.

	C	O	Na	Si	Cl	Cu	Sn	Pb
Outermost patina (A)	22.7	21.8	n.d.	1.2	4.4	27.4	19.1	3.4
Internal patina (B)	16.8	17.6	3.2	n.d.	n.d.	n.d.	n.d.	62.4
Internal patina (C)	17.2	n.d.	n.d.	n.d.	19.9	n.d.	n.d.	62.9

## Data Availability

Any data or material that support the findings of this study can be made available by the corresponding authors upon request.

## Supplementary Materials

The following supporting information can be downloaded at: https://www.mdpi.com/article/10.3390/ma17020442/s1, Figure S1: Selected XRD pattern for soil from Tharros; Figure S2: Selected XRD pattern for the soil from Sant’Antioco; Figure S3: Detail of the corrosion patina developed on the Ag-Cu7.5 disk; Figure S4: Stratigraphy of the corrosion patinas developed on the Ag-based alloys; Figure S5: Morphological appearance of the black arborescent compounds found on the bronze patina; Figure S6: Morphological features of the patina grown on the leaded bronze disk; Figure S7: Mineralogical assemblage of the corrosion patina grown on the bronze disk; Figure S8: Mineralogical assemblage of the corrosion patina grown on the leaded bronze disk; Figure S9: Mineralogical assemblage of the corrosion patina grown on the brass disk buried in the Sant’Antioco soil; Figure S10: Mineralogical assemblage of the corrosion patina grown on the brass disk buried in the Tharros soil; Figure S11: Structure of the Sn-enriched compound consisting of schoenfliesite; Figure S12: Structural and chemical features of the cross-sectioned patina grown on the bronze disk; Figure S13: Structural and chemical features of a Zn-enriched area on the cross-sectioned patina grown on the brass disk.
