# Supplementary material for: Early Stages of Metal Corrosion in Coastal Archaeological Sites: Effects of Chemical Composition in Silver and Copper Alloys
Source: Materials (Basel). 2024 Jan 17;17(2):442. doi: 10.3390/ma17020442 (PMC10820586; doi:10.3390/ma17020442)
Supplement: Supplementary file 1 [file materials-17-00442-s001.zip › materials-2786298-supplementary.pdf]

Supplementary material

Early Stages of Metal Corrosion in Coastal Archaeological Sites: Effects of Chemical Composition in Silver and Copper Alloys

Francesca Boccaccini [1,2,*], Cristina Riccucci [1], Elena Messina [1], Marianna Pascucci [1], Ferdinando Bosi [2], Luca Aldega [2], Alessandro Ciccola [3], Paolo Postorino [4], Gabriele Favero [3], Gabriel Maria Ingo [1] and Gabriella Di Carlo [1,*]

[1] Institute for the Study of Nanostructured Materials (ISMN), National Research Council (CNR), SP35d, 9, 00010 Montelibretti, Italy; cristina.riccucci@cnr.it (C.R.); elena.messina@cnr.it (E.M.); marianna.pascucci@cnr.it (M.P.); gabrielmaria.ingo@cnr.it (G.M.I.)

[2] Department of Earth Sciences, Sapienza University of Rome, Piazzale Aldo Moro, 5, 00185 Rome, Italy; ferdinando.bosi@uniroma1.it (F.B.); luca.aldega@uniroma1.it (L.A.)

[3] Department of Environmental Biology, Sapienza University of Rome, Piazzale Aldo Moro, 5, I-00185 Rome, Italy; alessandro.ciccola@uniroma1.it (A.C.); gabriele.favero@uniroma1.it (G.F.)

[4] Department of Physics, Sapienza University of Rome, Piazzale Aldo Moro, 5, I-00185 Rome, Italy; paolo.postorino@uniroma1.it

* Correspondence: francesca.boccaccini@uniroma1.it (F.B.); gabriella.dicarlo@cnr.it (G.D.C.)

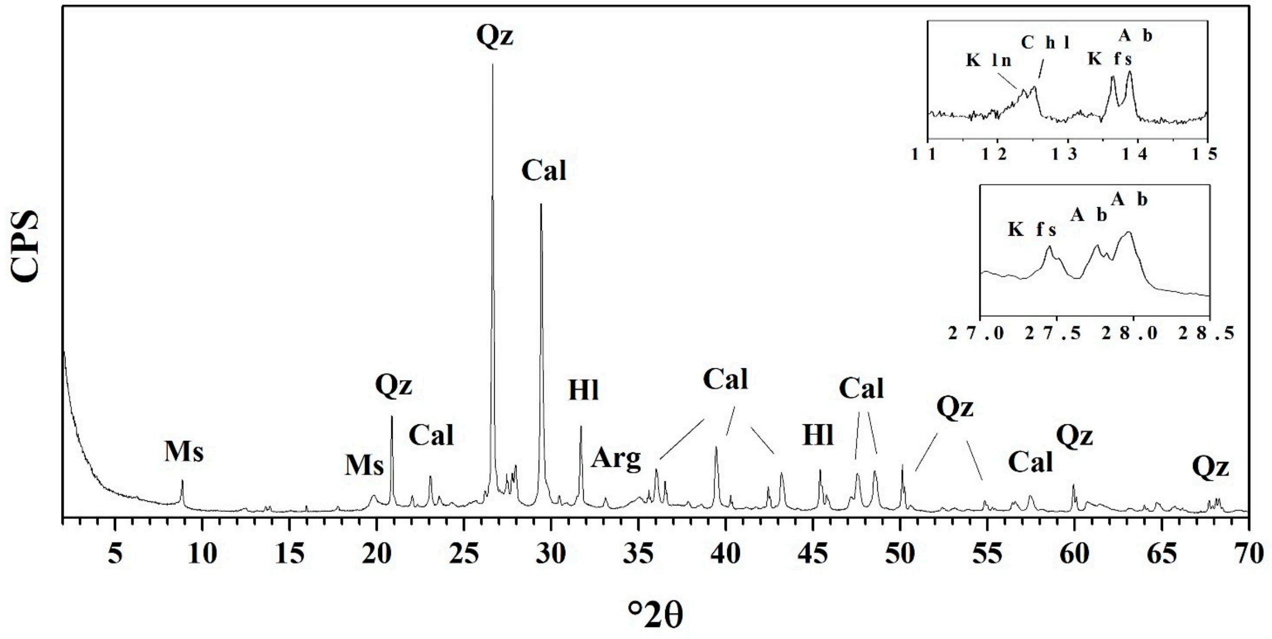

Figure S1. Selected XRD pattern for soil from Tharros. The mineralogical assemblage is characterized by quartz (Qz), calcite (Cal), aragonite (Arg), K-feldspar (Kfs), albite (Ab), muscovite (Ms), kaolinite (Kln), chlorite (Chl) and halite (Hl). The insets show a magnification of the diffractogram in the region from 11° to 15° 2θ and from 27° to 28.5° 2θ. Attributions were made using the following JCPDS codes: 46-1045 (Qz), 72-1652 (Cal), 71-2392 (Arg), 76-0830 (Kfs), 76-0927 (Ab), 86-1384 (Ms), 72-5860 (Kln), 07-0329 (Chl) and 05-0628 (Hl).

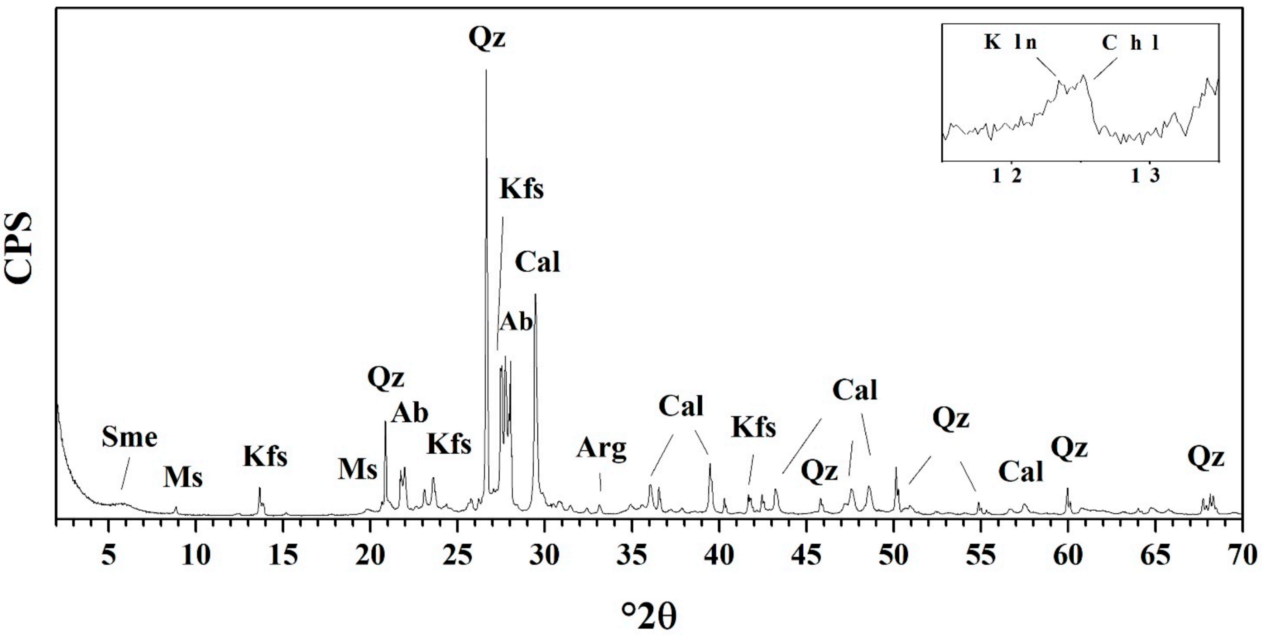

Figure S2. Selected XRD pattern for the soil from Sant'Antioco. The mineralogical assemblage is composed of quartz (Qz), calcite (Cal), aragonite (Arg), K-feldspar (Kfs), albite (Ab), muscovite (Ms), kaolinite (Kln), chlorite (Chl) and smectite (Sme). The inset shows a magnification of the diffractogram in the region from 11.5° to 13.5° 2θ. Attributions were made using the following JCPDS codes: 46-1045 (Qz), 83-1762 (Cal), 41-1475 (Arg), 83-1895 (Kfs), 76-0926 (Ab), 73-9857 (Ms), 72-5860 (Kln), 07-0329 (Chl) and 02-0014 (Sme).

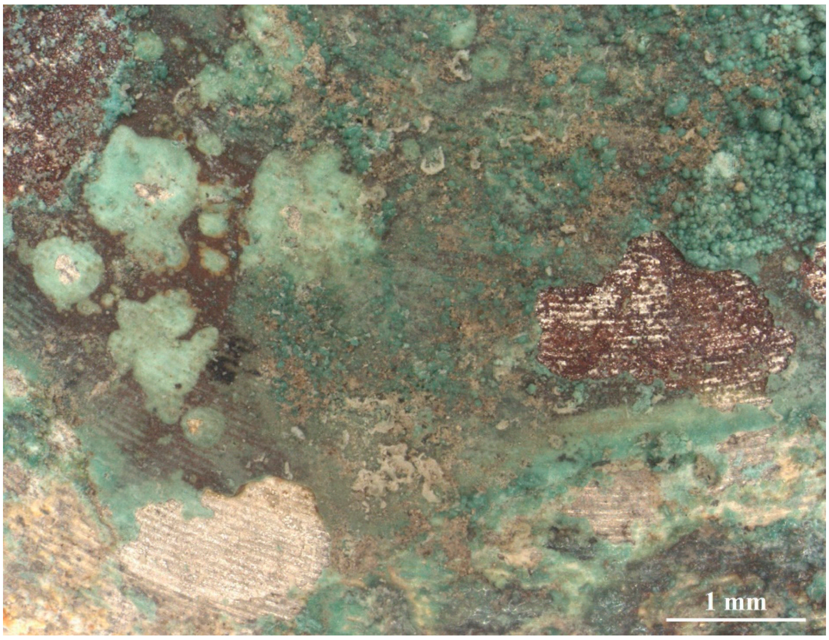

Figure S3. Detail of the corrosion patina developed on the Ag-Cu7.5 disk. The optical image shows green corrosion compounds over a layer of brown alteration products. Soil grains are clearly visible among the green mineral species.

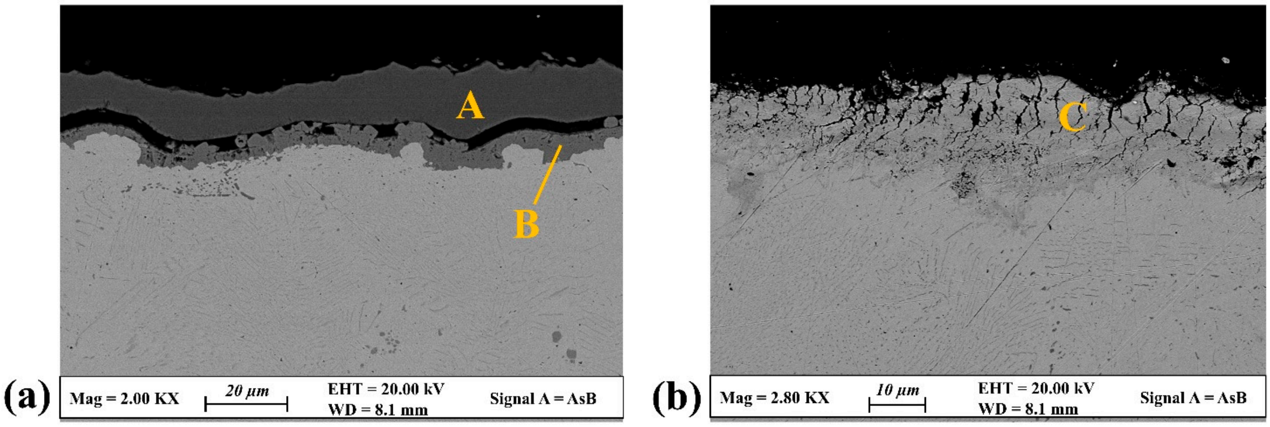

	O	Cl	Cu	Ag
A	48.5	19.7	31.8	n.d.
B	28.8	n.d.	71.2	n.d.
C	n.d.	17.5	n.d.	82.5

Figure S4. Stratigraphy of the corrosion patinas developed on the Ag-based alloys. FE-SEM backscattered images show the structure of the patina grown on the Ag-Cu7.5 disk (a) and on the Ag-Cu6.5 sample (b), while relative EDS analysis, acquired on spot areas of the cross-sections, reveals the chemical composition of each layer. Results are expressed in wt%; n.d. = not detected.

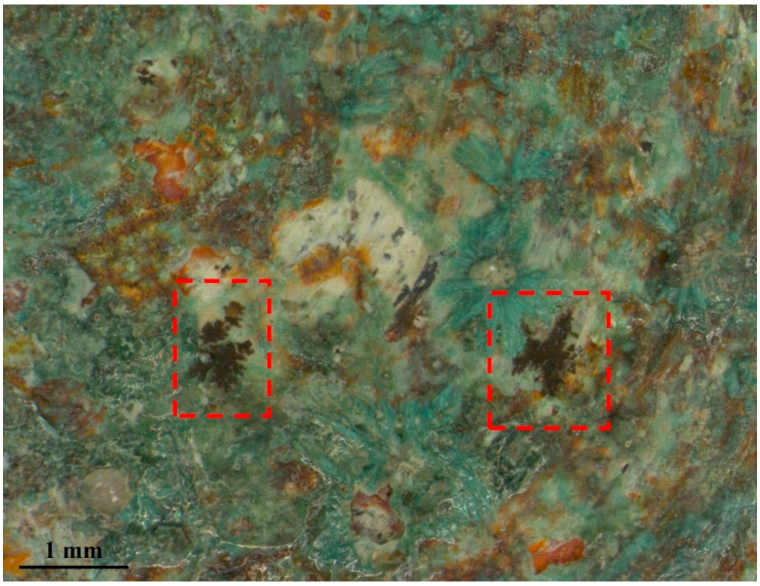

Figure S5. Morphological appearance of the black arborescent compounds found on the bronze patina.

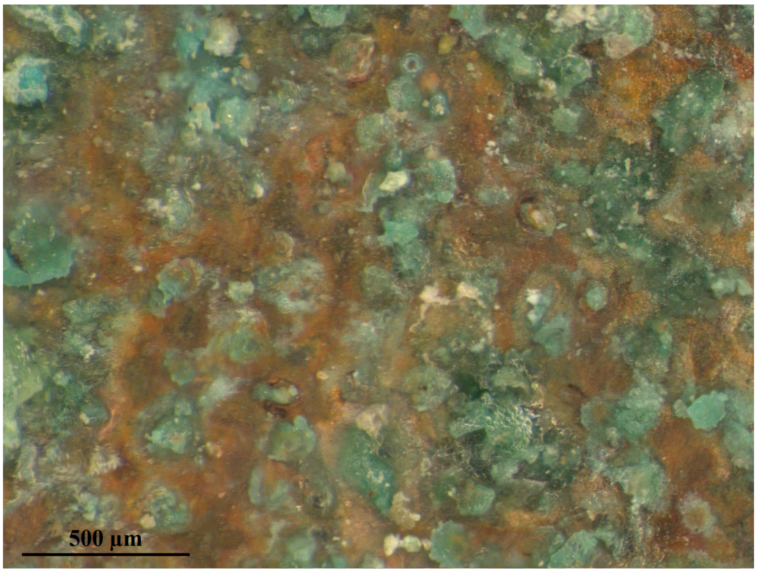

Figure S6. Morphological features of the patina grown on the leaded bronze disk. OM image shows the green compounds forming round shaped structures over an underlying orange layer.

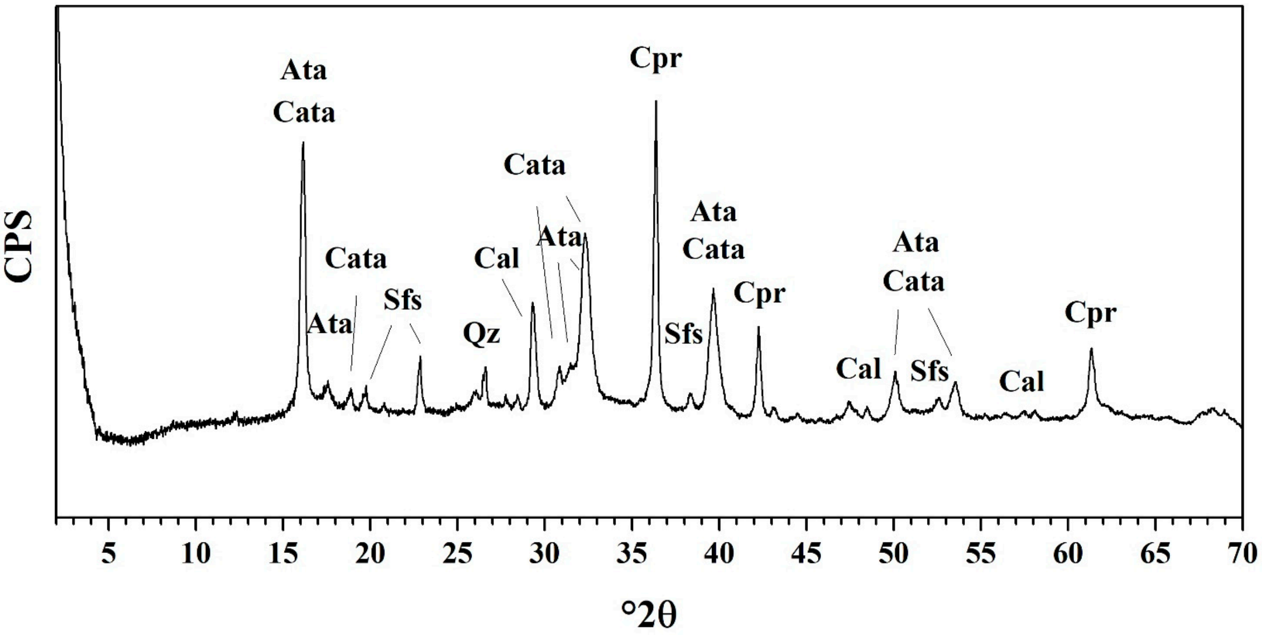

Figure S7. Mineralogical assemblage of the corrosion patina grown on the bronze disk. X-ray pattern acquired on the powder scratched from the surface shows cuprite (Cpr), atacamite (Ata), clinoatacamite (Cata), schoenfliesite (Sfs), calcite (Cal) and quartz (Qz). Attributions were made using the following JCPDS codes: 05-0667 (Cpr), 74-9208 (Ata), 50-1559 (Cata), 59-0542 (Sfs), 66-0867 (Cal) and 05-0490 (Qz).

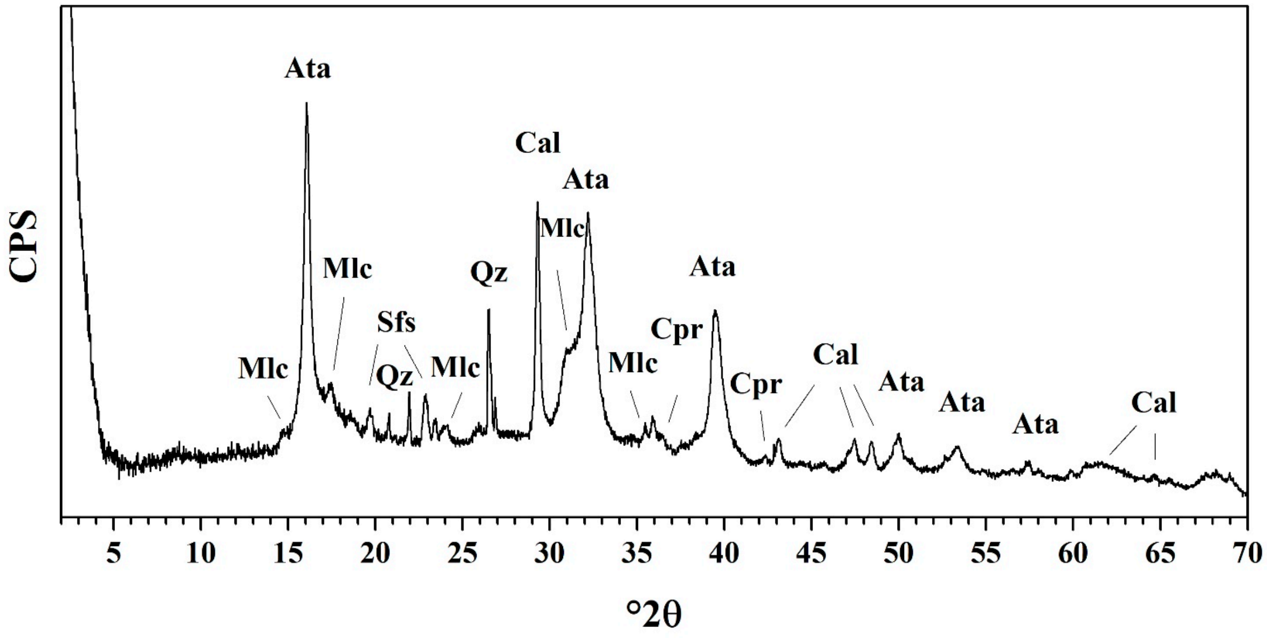

Figure S8. Mineralogical assemblage of the corrosion patina grown on the leaded bronze disk. X-ray pattern acquired on the powder scratched from the surface shows cuprite (Cpr), atacamite (Ata), malachite (Mlc), schoenfliesite (Sfs), calcite (Cal) and quartz (Qz). Attributions were made using the following JCPDS codes: 78-2076 (Cpr), 74-9208 (Ata), 56-0001 (Mlc), 09-0027 (Sfs), 66-0867 (Cal) and 46-1045 (Qz).

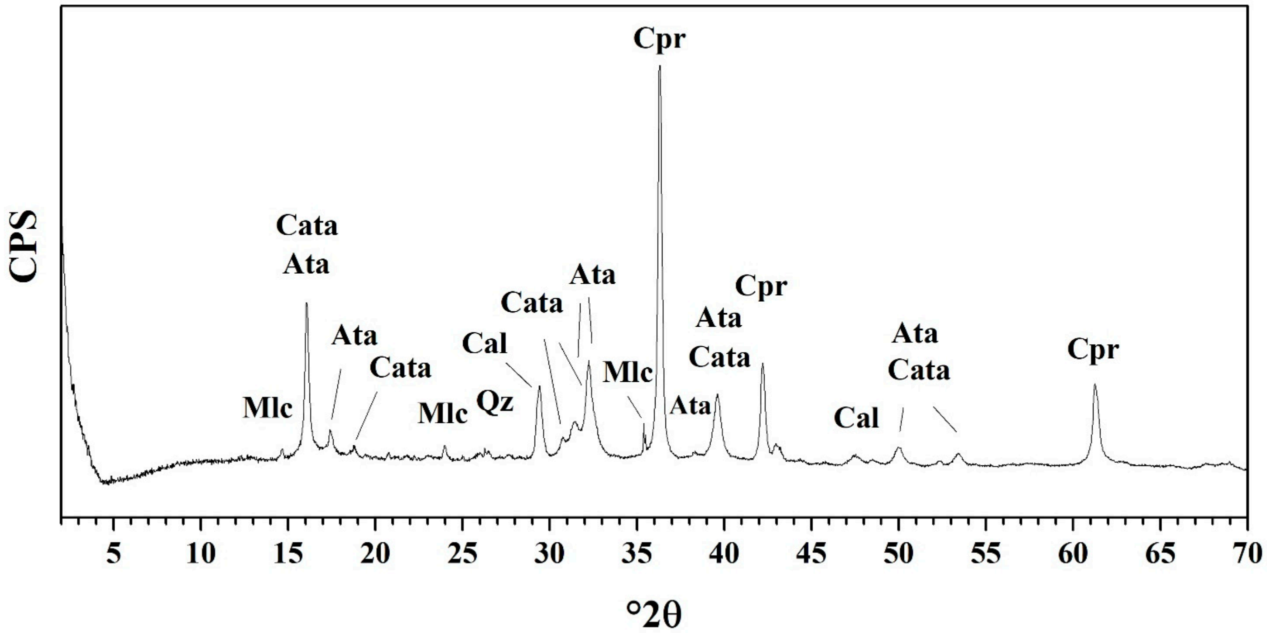

Figure S9. Mineralogical assemblage of the corrosion patina grown on the brass disk buried in the Sant'Antioco soil. X-ray pattern acquired on the powder scratched from the surface shows cuprite (Cpr), atacamite (Ata), clinoatacamite (Cata), malachite (Mlc), calcite (Cal) and quartz (Qz). Attributions were made using the following JCPDS codes: 78-2076 (Cpr), 25-0269 (Ata), 50-1559 (Cata), 41-1390 (Mlc), 05-0586 (Cal) and 46-1045 (Qz).

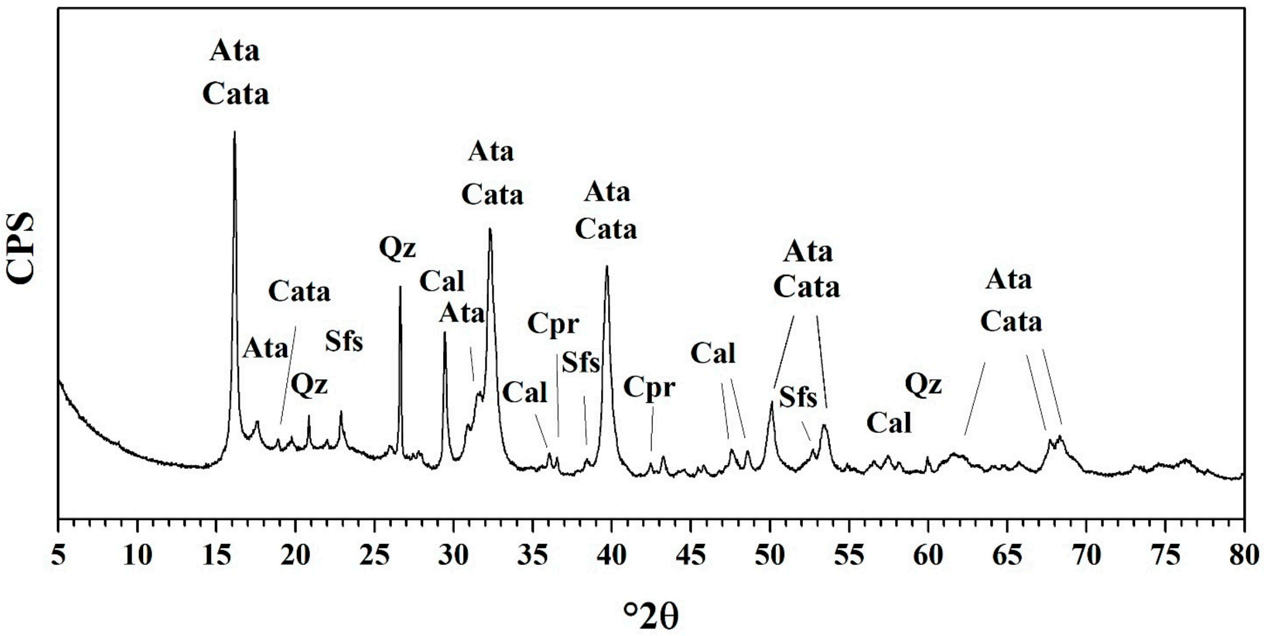

Figure S10. Mineralogical assemblage of the corrosion patina grown on the brass disk buried in the Tharros soil. X-ray pattern acquired on the powder scratched from the surface shows cuprite (Cpr), atacamite (Ata), clinoatacamite (Cata), schoenfliesite (Sfs), calcite (Cal) and quartz (Qz). Attributions were made using the following JCPDS codes: 78-2076 (Cpr), 25-0269 (Ata), 50-1559 (Cata), 09-0027 (Sfs), 05-0586 (Cal) and 46-1045 (Qz).

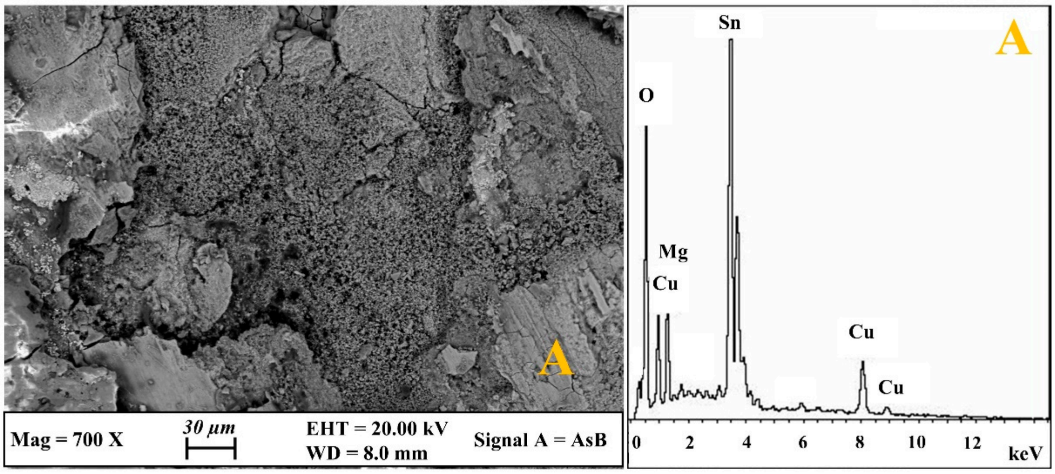

Figure S11. Structure of the Sn-enriched compound consisting of schoenfliesite. FE-SEM backscattered image and relative EDS spectrum show the morphology and the chemical composition of the flat and brittle layer of schoenfliesite developed on the bronze patina.

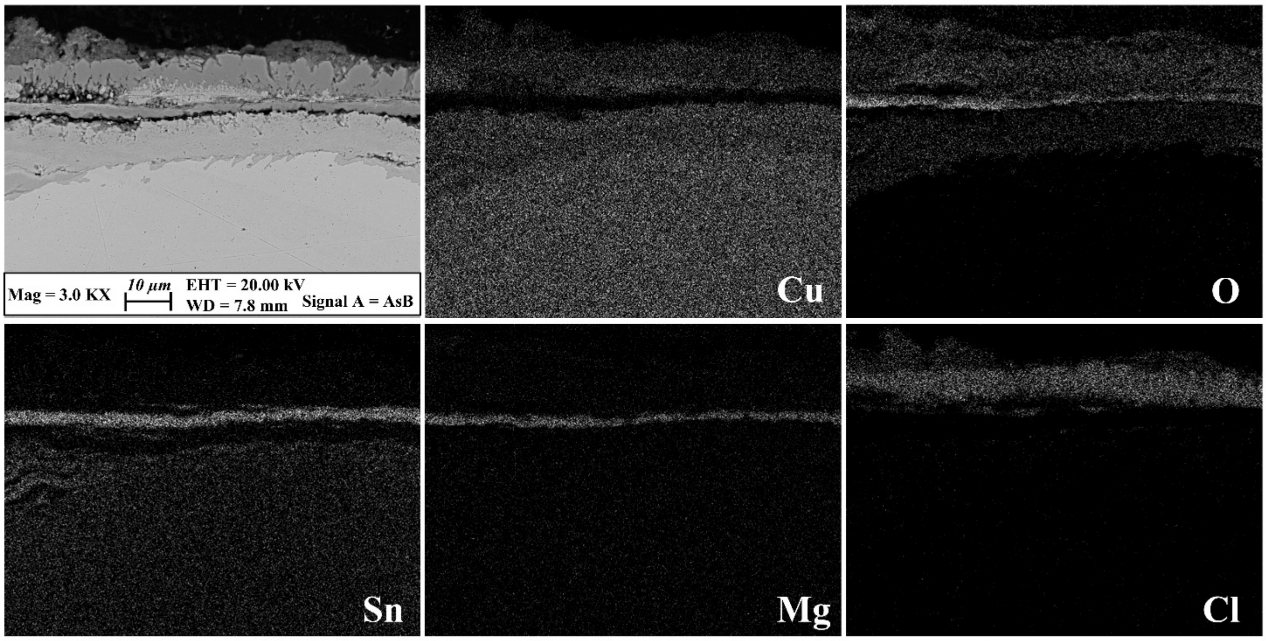

Figure S12. Structural and chemical features of the cross-sectioned patina grown on the bronze disk. FE-SEM backscattered image and relative X-ray elemental maps show a layered corrosion patina. The inner compounds consist of Cu and O, while a Sn and Mg enriched layer developed above these products marking the original surface bound. The outermost corrosion products are mainly Cu and Cl compounds.

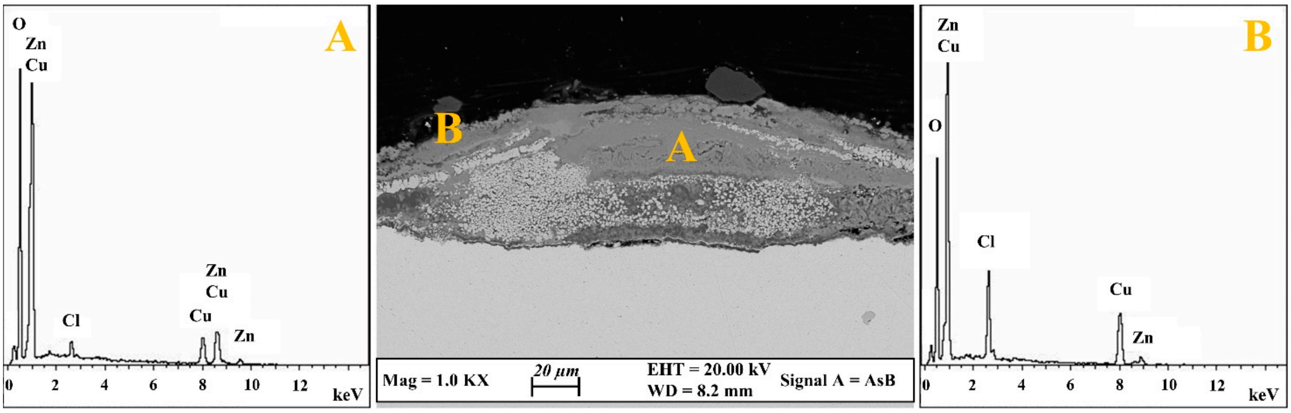

Figure S13. Structural and chemical features of a Zn-enriched area on the cross-sectioned patina grown on the brass disk. FE-SEM backscattered image and relative EDS spectra show an unusual enrichment in Zn, up to 30% by weight in the corroded layer (spectrum A). The external compounds are mainly copper hydroxychlorides (spectrum B).
